# *Lactobacillus johnsonii* N5 from heat stress-resistant pigs improves gut mucosal immunity and barrier in dextran sodium sulfate-induced colitis

**DOI:** 10.1016/j.aninu.2023.04.012

**Published:** 2023-08-04

**Authors:** Long Yuan, Chuyang Zhu, Fang Gu, Miaonan Zhu, Jiacheng Yao, Cuipeng Zhu, Shicheng Li, Kun Wang, Ping Hu, Yunzeng Zhang, Demin Cai, Hao-Yu Liu

**Affiliations:** a College of Animal Science and Technology, Yangzhou University, Yangzhou 225009, China; b Joint International Research Laboratory of Agricultural & Agri-Product Safety, The Ministry of Education of China, Yangzhou University, Yangzhou 225009, China; c Jiangsu Co-innovation Center for Prevention and Control of Important Animal Infectious Diseases and Zoonoses, Yangzhou University, Yangzhou 225009, China

**Keywords:** *Lactobacillus johnsonii*, Probiotic, Heat shock protein, Diarrhea, Heat stress, Treg/Th17

## Abstract

Developing effective strategies to prevent diarrhea and associated-gut disorders in mammals has gained great significance. Owing to the many health benefits provided by the commensal microbiota of the intestinal tract, such as against environmental perturbation, we explored the host phenotype-associated microbes and their probiotic potential. Based on the observations that the chronic heat stress-exposed weaned piglets present as heat stress-susceptible (HS-SUS) or heat stress-resistant (HS-RES) individuals, we confirmed the phenotypic difference between the two on growth performance (*P* < 0.05), diarrhea index (*P* < 0.001), intestinal heat shock protein 70 (HSP70) regulation (*P* < 0.01), and inflammatory responses (*P* < 0.01). By comparing the gut microbiome using 16S rRNA gene sequencing and KEGG functional analysis, we found that *Lactobacillus johnsonii* exhibited significantly higher relative abundance in the HS-RES piglets than in the HS-SUS ones (*P* < 0.05). Further experiments using a mouse model for chemical-induced inflammation and intestinal injury demonstrated that oral administration of a representative *L. johnsonii* N5 (isolated from the HS-RES piglets) ameliorated the clinical and histological signs of colitis while suppressing intestinal pro-inflammatory cytokines TNF-α and IL-6 production (*P* < 0.05). We found that N5 treatment enhanced tight junction proteins ZO-1 and occludin and cytoprotective HSP70 levels under physiological condition and restored their mucosal expressions in colitis (*P* < 0.05). In support of the high production of the anti-inflammatory cytokine IL-10, N5 promoted the intestinal Peyer's patches MHCII^+^ and CD103^+^ dendritic cell populations (*P* < 0.05), increased the regulatory T (Treg) cell numbers (*P* < 0.05), and decreased the Th17 population and its IL-17a production under physiological condition and during colitis (*P* < 0.01). Our results shed light on understanding the interaction between commensal *Lactobacillus* and the host health, and provide *L. johnsonii* N5 as an alternative to antibiotics for preventing diarrhea and intestinal diseases.

## 1 Introduction

Intestinal disorders, such as diarrhea, are clinical problems present in mammals at different life stages and cause adverse effects on productivity. In pigs, it causes high mortality (Casewell et al., 2003; Gresse et al., 2017; Hu et al., 2018; Lallès et al., 2007). Among the causes of diarrhea in pigs, environmental stress, such as dramatic changes in temperature, humidity, noise, management, nutrient availability, plays a major role (Buchet et al., 2017; Ringseis and Eder, 2022). When pigs are exposed to elevated temperatures during summer in sub-tropical and temperate regions, it can be detrimental to their growth performance, intestinal function, and health due to their deficiency of sweat glands (Gabler et al., 2018; Ringseis and Eder, 2022). Switching from suckling to feeding at weaning also represents a stressful event in life for pigs, which marks significant changes in the intestinal tract and the immune system, thereby inducing diarrhea and other diseases (Gresse et al., 2017; Lallès et al., 2007). The traditional method to prevent diarrhea in pigs is to add antibiotics to the diet (Lallès et al., 2007). However, the overuse of antibiotics in livestock has led to the spread of bacterial resistance and accumulation of antibiotic residues in animal products, threatening human health (Casewell et al., 2003; Liao and Nyachoti, 2017; Su et al., 2022). In addition, the long-term use of antibiotics in young animals also causes dysbiosis of the gut microbiota and immaturity of the immune system, resulting in antibiotic-associated diarrhea and increased morbidity (Casewell et al., 2003; Editorial, 2023). Therefore, developing effective strategies to prevent diarrhea and replace antibiotic usage in agriculture is urgently needed.

The mammalian gastrointestinal (GI) tract houses trillions of microorganisms. It is known that gut microbiota dysbiosis, characterized as reduced bacterial community diversity, increased growth of pathobionts, and/or loss of beneficial microbes, is closely related to the host's susceptibility to diseases (Gresse et al., 2017; Karasova et al., 2021). The inflammatory bowel disease (IBD) in humans is such an example (Heidarian et al., 2017; Ni et al., 2017; Samuel et al., 2007) and early weaning-induced stress and post-weaning diarrhea in piglets are other examples (Gresse et al., 2017; Hu et al., 2018; Su et al., 2022). In such a context, a targeted reconstitution of the disrupted bacterial community or supplementation of probiotics is emerging as promising approaches to combat GI disorders (Editorial, 2023). Probiotics are live microorganisms that can deliver health benefits to the host when given the appropriate species in an adequate amount (Swanson et al., 2020). Among bacterial taxa studied as potential probiotics, *Lactobacillus* spp. is a group of non-pathogenic, diverse commensal microbes within the intestinal tract, where multiple beneficial effects have been proven (Djukovic et al., 2022; Wang et al., 2020; Zheng et al., 2021; Zhu et al., 2022; Zuo and Marcotte, 2021). Using a preclinical mouse model of IBD, we have previously shown that oral administration of *Lactobacillus reuteri* R2LC improves intestinal integrity, inhibits inflammation (Ahl et al., 2016; Liu et al., 2022), and regulates the B cell-IgA-microbiota interactions at the Peyer's patches (PP) (Liu et al., 2021). Furthermore, it is demonstrated that *Lactobacillus gasseri* LA39 and *Lactobacillus frumenti* mediate diarrhea resistance in early-weaning piglets (Hu et al., 2018). In addition, *Lactobacillus johnsonii* and its metabolites have been shown to increase anti-inflammatory cytokine IL-10 production by dendritic cells (DC) and promote regulatory T (Treg) cell differentiation in vitro (Zheng et al., 2021). However, the mechanism behind these probiotic regulations is far from precise, and which bacterial species of the microbiota contribute most to the clinical success warrant further explorations.

Indeed, the gut microbiota interacts with the host through a multi-layer system of the intestinal mucosa, including secreted anti-bacterial agents, epithelium, and innate and adaptive immune cells (Maynard et al., 2012). In addition to gut microbiota dysbiosis, the disruption of intestinal epithelium tight junctions (TJ) represents the breaking down of the first line of defense against various challenges (Ulluwishewa et al., 2011), including decreased expressions of vital TJ proteins ZO-1 (Ibrahim et al., 2020), claudins (Sayoc-Becerra et al., 2020) and occludin (Lin et al., 2019), as well as junctional adhesion molecules (JAM) such as JAM-A (Ahl et al., 2016). In contrast, data from animal studies suggest that TJ functioning can be restored by specific beneficial bacteria and/or their products (Ahl et al., 2016; Akbari et al., 2015; Wang et al., 2018a). Furthermore, we and others have also addressed the importance of cytoprotective heat shock proteins (HSP) and reported associations between HSP expression and the relative abundance of *Lactobacillus* spp. in maintaining gut homeostasis (Liu et al., 2014a, 2014b, 2022; Otaka et al., 2006). Continuing to delineate clear targets of probiotics in the highly stratified mucosal system, including immune components, is important for managing intestinal diseases.

In the current study, we, therefore, aimed to identify close associations between specific commensal microbes and host health. We observed differences in growth performance, diarrhea index, intestinal HSP70 regulations, and inflammation of piglets exposed to the same cyclic heating regimen. They were assigned as heat stress-susceptible (HS-SUS) and heat stress-resistant (HS-RES) individuals. We found that the relative abundance of *L. johnsonii* is significantly higher in the HS-RES piglets than in those who had diarrhea and subsequently isolated a representative strain N5 from *L. johnsonii*. Finally, we revealed the protective effects of *L. johnsonii* N5 against dextran sulfate sodium (DSS)-induced colitis and its roles in strengthening intestinal barrier function and balancing the response of intestinal innate and adaptive immunity.

## 2 Materials and methods

### 2.1 Animal ethics

All animal procedures, including mice and pigs used in this study, were approved by the Animal Care and Use Committee of Yangzhou University under the ethical permit numbers, YZUDWSY 2017-09-06 and SYXK (Su) IACUC 2021-0026, respectively.

### 2.2 Pig trial

A total of 18 early-weaned male Meishan piglets were used. They were obtained from a large animal experiment from our group, raised on a pig breeding farm (Nanjing, China). Each piglet was housed individually and received no antibiotics from birth till the end of the experiment. They were fed a corn-soybean meal-based diet (Table 1) formulated according to swine nutrition requirements as recommended by National Research Council (NRC, 2012). The feed ingredient and diet samples were taken. The crude protein concentrations were determined using Kjeldahl automated apparatus (K9805, Shanghai Analytical Instrument Co., Ltd., Shanghai, China) according to AOAC methods 976.06 (AOAC, 2007); the mineral contents (P and Ca) were analyzed by the 5110 ICP-OES (Agilent Technologies Australia (M) Pty. Ltd., Australia) as described previously (Huang et al., 2009); and lysine, methionine and threonine were determined using HPLC (Water HPLC system, Water Corporation, MA, USA) as described previously (Guay et al., 2006). Twenty piglets at the age of 35 days were subjected to cyclic heating treatment (36 °C, 12:00 to 16:00; 20 °C, 16:00 to 12:00) at 40% to 60% humidity for 8 weeks, using automatic heaters (175W Heat preservation lamp, MULHE, Henan, China) to induce chronic heat stress. Whereas the healthy control group (*n* = 6) was raised at a temperature of 26 to 28 °C. During the experiment, body weight and diarrhea index were recorded weekly. Subsequently, 12 out of 20 piglets that received the heating treatment were identified as HS-RES or as HS-SUS individuals based on their performance, diarrhea scores, and inflammatory responses in circulation and were sampled at the end of the experiment.

**Table 1:** Ingredient composition and nutrient content of the experimental diet (%, as-fed basis).

Ingredients	Content	Nutritional levels	Content
Corn	45.50	Digestible energy^2^, Mcal/kg	3.20
Barley	18.00	Crude protein^3^	16.91
Soy bean meal (dehulled)	20.00	Total phosphorus^3^	0.60
Corn gluten meal	6.00	Calcium^3^	0.78
Wheat shorts	5.00	Lysine^3^	1.11
Soybean oil	1.50	Methionine^3^	0.38
Premix^1^	4.00	Threonine^3^	0.65

1 Content per kilogram premix: Ca 3.77 g, P 1.02 g, Mg 3.10 g, K 1.50 g, Na 0.37 g, Cl 0.11 g, S 84.00 g, Fe 120,493 mg, Cu 29,791 mg, Co 52.62 mg, Mn 24,076 mg, Zn 134 mg, I 238 mg, Se 476 mg, retinol 5,952,000 IU, cholecalciferol 595,200 IU, α-tocpherol 101,190 mg, thiamin 2,381 mg, riboflavin 5,952 mg, pyridoxine 3,750 mg, vitamin B_12_ 24 mg, pantothenic acid 11,914 mg, nicotinic acid 23,810 mg, biotin 2,238 mg.

2 Calculated value.

3 Values determined by analysis; each value is based on triplicate determinations.

### 2.3 Diarrhea index measurement

The diarrhea index was evaluated to assess the severity of intestinal inflammation. Experimental animals were inspected for diarrhea, and fresh droppings were scored immediately as follows: 0 = normal feces (solid in shape); 1 = moist feces (semi-solid); 2 = mild diarrhea (loose feces, deformed); 3 = severe diarrhea (liquid feces) as previously described (Atarashi et al., 2013; Hu et al., 2018).

### 2.4 Microbiota composition analysis and bacterial isolation

Genomic DNA was extracted from the feces (0.2 mg) using the TIANamp Stool DNA Kit (TIANGEN, Beijing, China) according to the protocols of the manufacturer. The DNA concentration was determined using a Nano-Drop 1000 spectrophotometer (F-3100, Suizhen, Hangzhou, China). Forward primer 515F (GTGCCAGCMGCCGCGGTAA) and reverse primer 806R (GGACTACNNGGGTATCTAAT) were used to amplify the V4 region of the bacterial 16S ribosomal RNA gene (95 °C for 3 min, followed by 30 cycles at 95 °C for 45 s, 56 °C for 45 s, 72 °C for 45 s, and a final extension at 72 °C for 10 min). Purified amplicons were pooled in equimolar and paired-end sequenced (2 × 250 bp) on an Illumina platform according to the standard protocols. Primers, low-quality sequences, and barcode sequences were removed from the raw reads. The contralateral clean reads were merged using FLASH (Baltimore, MD, USA, version 1.2.11) with a minimum overlap of 10 bp and a mismatch error rate of 2%. Operational taxonomic unit (OTU) with 97% similarity were identified using the UPARSE (version 9.2.64) pipeline. Bacterial diversity was estimated for the observed species, Shannon, and Simpson indexes using the MOTHUR program (version 1.35.0). Heatmap generation and the partial least squares discriminant analysis (PLS-DA) were performed on the relative abundance of OTU. Microbial gene function was predicted using the PICRUSt (version 2.1.4) software and annotated using the Kyoto Encyclopedia of Genes and Genomes (KEGG) database (Caporaso et al., 2010; Langille et al., 2013). The 16S sequencing data in the present study was uploaded to GenBank at the National Center for Biotechnology Information with accession number PRJNA929297.

For bacterial isolation and preparation, fresh feces were collected using sterile tubes from the healthy control and piglets exposed to heat stress. Only the central inner layer of the feces was collected using aseptic sticks to prevent potential contamination from either the soil or air. They were pooled and prepared for isolation. Briefly, 1 g of sample was homogenized with 9 mL of 9 g/L de Man, Rogosa, and Sharpe (MRS) medium to make an initial dilution (1 × 10^−1^) and incubated in an anaerobic atmosphere at 37 °C for 24 h. Serial dilutions were made for the overnight cultured samples, and 100 μL of the appropriate dilutions (1 × 10^−3^, 1 × 10^−4^, and 1 × 10^−5^) were spread on plates in triplicate on MRS medium (Oxoid), and incubated in an anaerobic atmosphere at 37 °C for 24 h. Distinct colonies were isolated, purified, and sub-cultured more than thrice, and the purity of the obtained strains were verified by microscope examination. DNA was extracted from pure cultures, and the 16S rDNA sequences were amplified using the 27F (5′-AGAGTTTGATCCTGGCTCAG-3′) and 1492R (5′-TACGGCTACCTTGTTACGACTT-3′) primer set, and the amplicon products were sequenced (Suzhou Jinweizi Biotechnology Co., Ltd., Suzhou, China). The taxonomic affiliation of the isolates was then determined using the EzBioCloud database (Yoon et al., 2017). The maximum-likelihood phylogenetic tree was constructed using FastTree version 2.1.7 (Price et al., 2010). Finally, a *L. johnsonii* N5 isolate was obtained from the feces of HS-RES piglets using the corresponding medium to study its probiotic potential in vivo. In addition, its sequencing read data have been deposited in the National Center for Biotechnology Information GenBank database under the accession number OQ256705.

### 2.5 Mice and DSS-induced colitis

Eight-week-old BALB/c mice (male) were purchased from Yangzhou University. Mice were housed under standardized conditions (21 to 22 °C, 12-h light and 12-h dark cycle) in specific-pathogen-free (SPF) facility for 1 week for acclimation. Thereafter animals with similar body weights were randomly assigned to the following 4 treatment groups: the control, the pre-treatment of *L. johnsonii* N5 (N5), the DSS group, and the co-treatment of *L. johnsonii* N5 with DSS (N5-DSS). For the N5 group, *L. johnsonii* N5 was freshly prepared and was given to mice per-orally at 10^8^ CFU per mouse per day for 7 consecutive days. Dextran sodium sulfate-mediated colitis was induced by administration of 2.5% (wt/vol) DSS (MW 36,000 to 50,000, Yeasen, Shanghai, China) in drinking water for 7 consecutive days, while disease severity was cumulatively scored as the disease activity index (DAI) including body weight loss, stool consistency, and blood content as previously described (Ahl et al., 2016). For the N5-DSS group, *L. johnsonii* N5 was given daily for 14 days starting 7 days prior to the 2.5% DSS-treatment. Mice from the control group were fed a commercial chow diet (MD17121, Medicience, Jiangsu, China) and received vehicle treatment when appropriate.

### 2.6 Collection of samples

All animals were euthanized humanely at the end of the experiment for sampling. EDTA was pre-loaded into blood collection tubes, and blood was taken from the jugular vein of pigs. Plasma was obtained by centrifuging blood samples at 2000 × *g* for 20 min at 4 °C. After the opening of the abdomen, colonic tissues and fecal samples were collected, immediately frozen in liquid nitrogen, and preserved at −80 °C until further analysis.

The mice were sacrificed after anesthesia. Blood samples were collected via cardiac draw into tubes with pre-loaded EDTA, and plasma was obtained by centrifugation of blood samples at 2000 × *g* for 20 min at 4 °C, and then stored at −80 °C until analysis. The colonic tissues and fecal samples were collected sterilely and stored at −80 °C for further analysis. Moreover, pieces of colonic tissues (approximately 0.5 cm) were obtained and processed for histology and immunofluorescent microscopy. In addition, intestinal lymphoid tissues were freshly harvested and prepared as single-cell suspensions for flow cytometry.

### 2.7 ELISA analyses

For the porcine samples, tumor necrosis factor-α (TNF-α), interleukin (IL)-6, IL-10, and chemokine (C–X–C motif) ligand 1 (CXCL1) were measured using enzyme-linked immunosorbent assay kits (Mlbio, Shanghai, China) according to the manufacturer's instructions. The concentrations of TNF-α, IL-6, interferon gamma (IFN-γ), and IL-10 were measured in the colon of mice using mice ELISA kits (R&D Systems, Minnesota, USA), according to the manufacturer's instructions. Tissues were homogenized and measured for protein concentration by the bicinchoninic acid (BCA) method, and values were normalized to tissue protein content. In addition, plasma levels of IL-10 in mice were determined using ELISA kit (MultiScience, Hangzhou, China) according to the manufacturer's instructions.

### 2.8 Quantitative real-time PCR

Total RNA was extracted from colonic tissues using a Trizol reagent (Thermo Fisher Scientific, Massachusetts, USA) according to the manufacturer's instruction. The purity and total RNA were determined by Nanodrop1000 (F-3100, Suizhen, Hangzhou, China). The cDNA was synthesized from 1 μg of total RNA by reverse transcription kits (Vazyme Biotech Co., Ltd., Nanjing, China). Using SYBR green PCR mix (Vazyme Biotech Co., Ltd., Nanjing, China) on a QuantStudio3 instrument (A28567, Thermo Fisher Scientific, Massachusetts, USA) and the parameters were as follows: 95 °C for 5 min, 40 cycles of 95 °C for 10 s, and 60 °C for 30 s, 72 °C for 30 s, 1 cycle of 95 °C for 15 s, 60 °C for 60 s, 95 °C for 15 s. The *GAPDH* expression was used as the internal control. The 2^−ΔΔCT^ method was used to calculate the relative expression of target genes compared to *GAPDH*. The primers used for qPCR are listed in Table S1.

### 2.9 Western blotting analysis

Intestinal tissues were lysed with 500 μL cell lysis buffer for Western blotting (Biosharp, Hefei, China) supplemented with phosphatase and protease inhibitor (Beyotime, Nanjing, China) according to the manufacturer's instructions. The tissue debris was removed by centrifugation at 10,000 × *g* for 10 min at 4 °C. Cellular proteins in the supernatant were collected and were quantified using an Enhanced BCA Protein Assay Kit (Beyotime, Shanghai, China). Adding 7 μL of total 23 μg of sample protein into the glue hole and the protein was separated in the electrophoresed gel. Next, the separated proteins were transferred to polyvinylidene fluoride (PVDF) membranes (0.45 μm, Merck, CA, USA). After being blocked with 5% skim milk at room temperature (RT) for 2 h, the samples were incubated with primary antibodies overnight at 4 °C. After washing 3 times with cold PBS and incubating with a secondary antibody at RT for 2 h, the membranes were visualized using an ECL detection reagent using a Tanon 5200 Multi imaging system (Tanon 5200, Tanon, Shanghai, China). For the detection, primary antibodies against HSP70 (bs-0244R, Bioss, Beijing, China), HSP27 (bs-0730R, Bioss, Beijing, China) and β-actin (sc-47778, Santa Cruz Biotechnology, New Mexico, USA), and the corresponding secondary antibodies (goat anti-rabbit IgG HRP, HX-2031, or goat anti-mouse IgG HRP, HX-2032; Huaxingbio, Beijing, China) were used.

### 2.10 Histological analysis

Tissues from the distal colon of mice were routinely fixed in 4% paraformaldehyde overnight, dehydrated in 70% ethanol, and embedded in paraffin. They were sectioned (5 μm in thickness), stained with hematoxylin and eosin (H&E), and were observed with a light microscope (Leica DMi8, Leica Corp, Wetzlar, Germany). Subsequently, duplicate slides per mouse were used for histological analysis. One individual blindly evaluated all slides using a scoring system as previously described (Cooper et al., 1993). In brief, the crypt damage was determined from 0 to 4 with 0 being intact and 4 the entire crypt and epithelium lost, multiplied by the percentage of the tissue damages.

### 2.11 Immunohistochemistry

The colonic tissue samples of mice were embedded in Tissue-Tek O.C.T. and snap-frozen in liquid nitrogen. Thereafter, the samples were cryo-sectioned (10 μm in thickness) and stained for target proteins. Antibodies raised against the following mouse antigens were used as follows: ZO-1 (ABIN602576, antibodies-online, Aachen, Germany), occludin (ABIN1108503, antibodies-online), JAM-A (AF1077, R&D Systems, Minnesota, USA), and HSP70 (ADI-SPA-812, Enzo, New York, USA). Accordingly, secondary antibodies were used to amplify signals with Alexa Fluor 647 anti-guinea pig, Alexa Fluor 488 anti-rat, goat anti-rat-IgG-AF555, and Alexa Fluor 488 anti-rabbit IgG, respectively. Nuclei were counterstained with Hoechst 33342 (Thermo Fisher Scientific, Massachusetts, USA). Two slides of each mouse (*n* = 6 per group) were imaged using a confocal Laser Scanning Microscope (Leica CS SP2, Wetzlar, Germany), and mean fluorescence intensity (MFI) was quantified using ImageJ software (version 1.8.0).

### 2.12 Flow cytometry

Single-cell suspensions from PP and mesenteric lymphoid nodes (used as staining controls) were prepared by mashing tissues through 40-μm cell strainers in PBS containing 0.05% fetal bovine serum (FBS) and 2 mM EDTA. The samples were blocked with anti-CD16/32 (2.4G2, BD Bioscience, New Jersey, USA), stained with antibodies anti-CD3 (17A2) and CD4 (GK1.5) from BioLegend (CA, USA), anti-CD8α (5H10, Thermo Fisher Scientific, Massachusetts, USA), and anti-CD11c (N418), MHC Class II (M5/114.15.2) and CD103 (2E7) from eBioscience (CA, USA). For intracellular staining of forkhead box protein P3 (FoxP3) (MF-14), retinoic acid receptor-related orphan receptor gamma t (RORγt) (AFKJS-9), and IL-17a (TC11-18H10.1), cells were fixed and permeabilized using True-Nuclear Transcription Factor Buffer Set according to the manufacturer's instructions (BioLegend, CA, USA). Data were acquired on a CyAn ADP7 flow cytometer (CyAn ADP7, Beckman Coulter, CA, USA) and analyzed with FlowJo version 10.0.8 (Tree Star, Inc., CA, USA).

### 2.13 Statistical analysis

Statistical analysis was performed using GraphPad Prism version 9 (GraphPad Software, Inc., CA, USA). Two-tailed Student's *t*-test was used to directly compare two groups, and one-way analysis of variance (ANOVA) with Tukey's post hoc test was used to compare all groups. The difference in body weight, diarrhea index, and DAI was calculated as an area under the curve. VEGAN package version 2.0-7 was used for the multivariate analysis of the microbiota data. Data were presented as mean ± SEM., and the number of animals per group was indicated in each figure legend. *P*-value < 0.05 was considered significant.

## 3 Results

### 3.1 The phenotypic distinction between the HS-SUS and HS-RES piglets

**Fig. 1:**
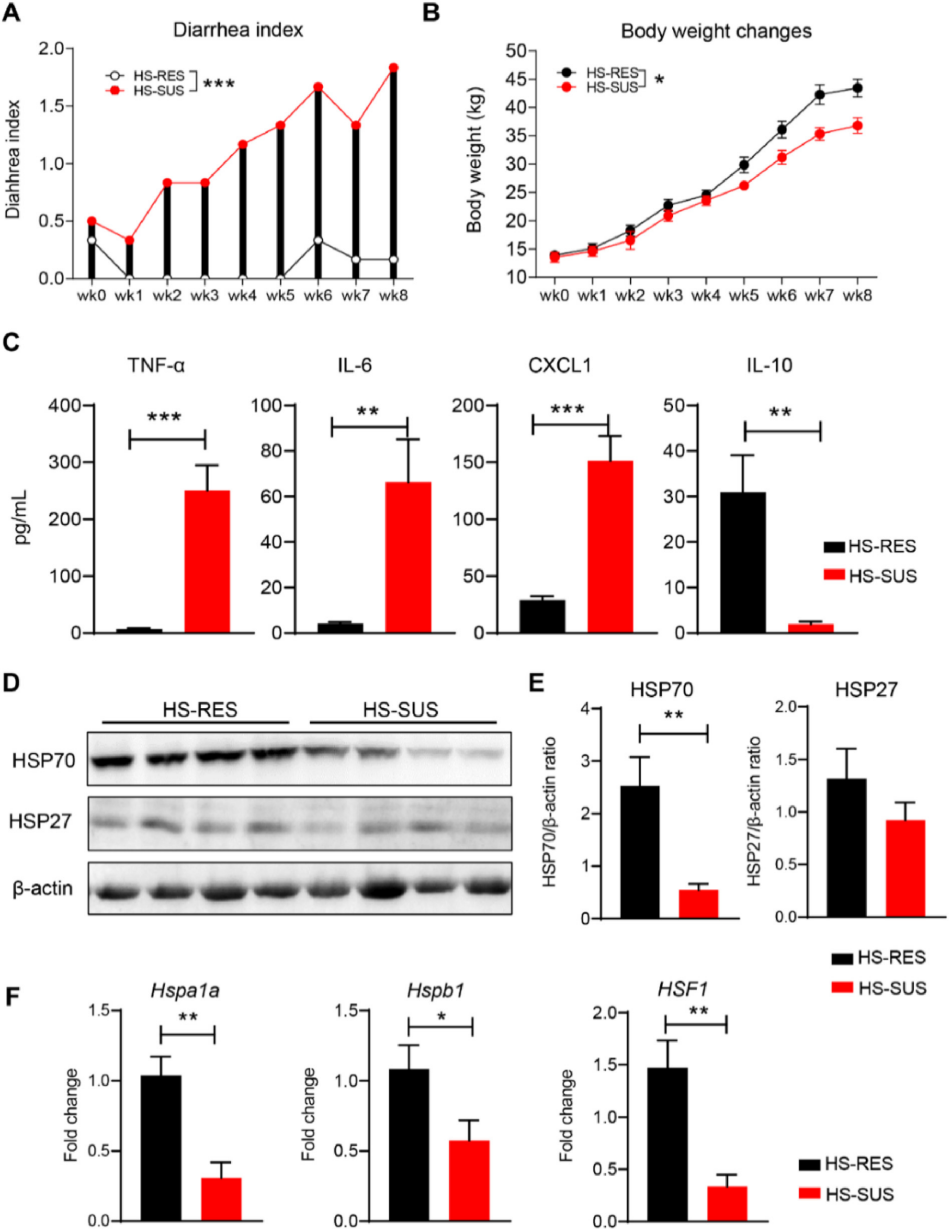
The phenotypic difference between the heat stress-susceptible and heat stress-resistant piglets in growth performance, inflammatory responses, and gut physiology. (A and B) The diarrhea indexes and body weight of Meishan piglets were recorded weekly during 8 weeks of cyclic heating treatment. The difference of body weight, diarrhea index was calculated as area under the curve. (C) The levels of TNF-α, IL-6, CXCL1 and IL-10 in porcine plasma. (D) Western blots showing the levels of HSP70, HSP27 and β-actin in the colonic tissues of HS-RES and HS-SUS pigs. (E) The expression of HSP70 and HSP27 normalized to β-actin expression. (F) qRT-PCR analysis of genes expression (fold change) of *Hspa1a, Hspb1, HSF1* in the colonic tissues. HS-RES = heat stress resistant; HS-SUS = heat stress susceptible; TNF-α = tumor necrosis factor-alpha; IL-6 = interleukin-6; CXCL1 = chemokine (C–X–C motif) ligand 1; IL-10 = interleukin-10; HSP27 = heat shock proteins 27; HSP70 = heat shock proteins 70; *Hspa1a* = heat shock protein family A (HSP70) member 1A; *Hspb1* = heat shock protein family B (small) member 1; *HSF1* = heat shock factor 1. Data are presented as the means ± SEM, *n* = 6 piglets per group. ∗*P* < 0.05, ∗∗*P* < 0.01, ∗∗∗*P* < 0.001, using the two-tailed Student's *t*-test.

Based on the observations that the chronic environmental stress-exposed weaned piglets naturally occurred as HS-SUS or HS-RES individuals, we studied changes in their growth performance, inflammatory responses, and gut physiology. We found that compared with the HS-SUS group, the HS-RES piglets resisted having diarrhea throughout the heating treatment period, reflected by the significantly lower diarrhea index (*P* < 0.001; Fig. 1A). Consistently, the body weight of the HS-RES animals remained higher than that in the HS-SUS group (*P* < 0.05; Fig. 1B). Furthermore, compared with the HS-SUS group, plasma concentrations of pro-inflammatory cytokine/chemokine TNF-α, IL-6, and CXCL1 were significantly lower, whereas the anti-inflammatory cytokine IL-10 was significantly higher in the HS-RES group (*P* < 0.01; Fig. 1C). The Western blotting analysis revealed that the colonic HSP70 expression was markedly suppressed in the HS-SUS piglets compared with the HS-RES group (*P* < 0.01; Fig. 1D and E). In contrast, the expression levels of HSP27 did not differ between groups (*P* > 0.05; Fig. 1D and E). In addition, qRT-PCR analysis was conducted and showed that the expressions of heat shock protein family A (HSP70) member 1A (*Hspa1a*, encoding HSP70), heat shock protein family B (small) member 1 (*Hspb1*, encoding HSP27), and their transcription factor *HSF1* in the colon of HS-SUS piglets were all significantly down-regulated when compared with the HS-RES group (*P* < 0.05; Fig. 1F).

### 3.2 The gut microbiome differences between the HS-SUS and HS-RES piglets

**Fig. 2:**
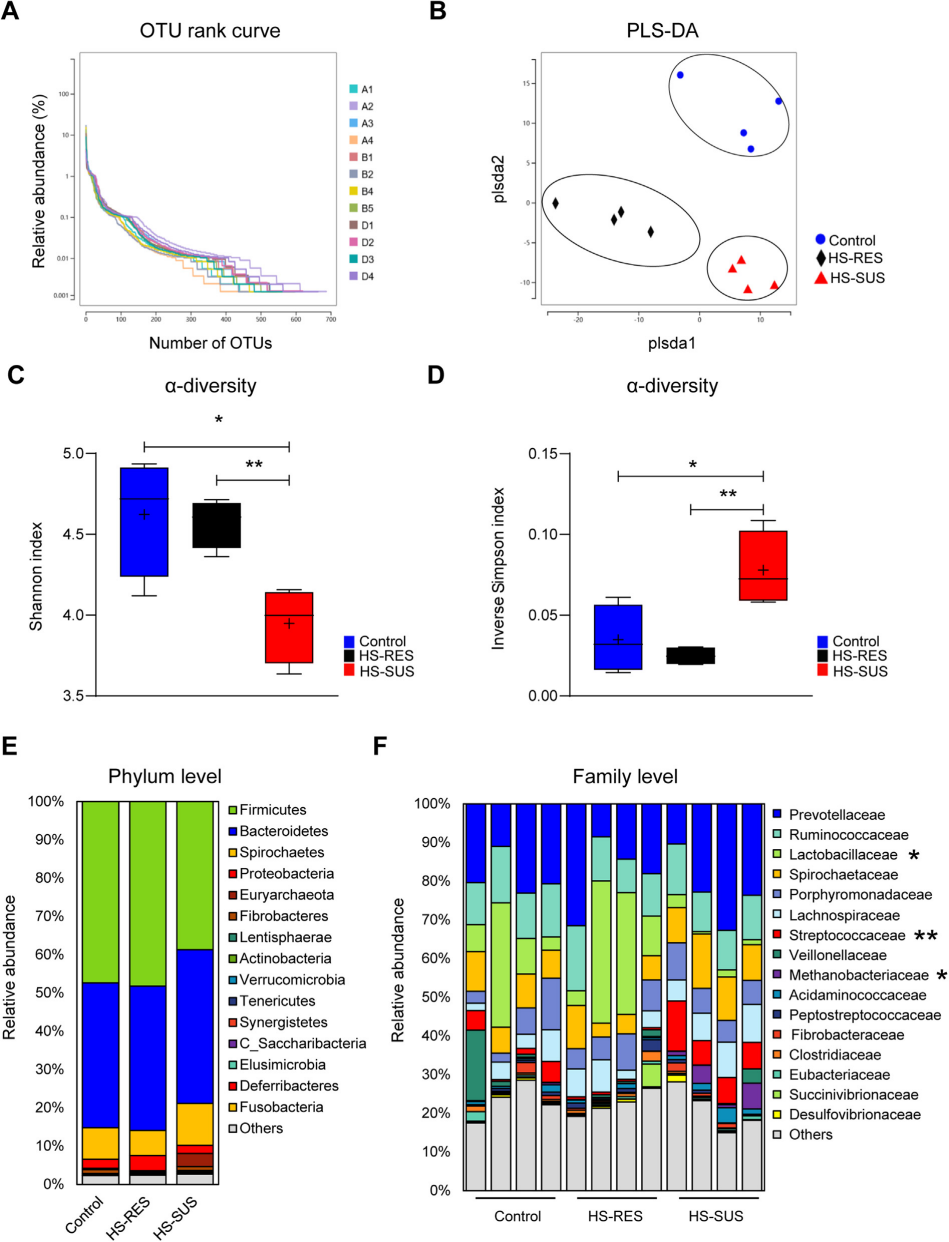
The gut microbiota of the heat stress-susceptible, the heat stress-resistant and the healthy control piglets were compared using 16S rRNA gene amplicon sequencing. (A) The rank abundance curves of 12 fecal microbiota samples including piglets from the control group received no heating treatment, reflected the abundance and rarity of OTU in the bacterial community. (B) PLS-DA, (C) Shannon index and (D) inverse Simpson index were used to analyze the overall microbial community. Data are presented as median values. (E) Average relative abundance of bacterial taxa at phylum level. (F) Bar graph depicts bacterial community composition of individual pigs at family level, *n* = 4 per group. HS-RES = heat stress resistant; HS-SUS = heat stress susceptible; OTU = operational taxonomic units; PLS-DA = partial least squares discriminant analysis. ∗*P* < 0.05, ∗∗*P* < 0.01.

Gut microbiota plays important roles in physiological health, while exposure to environmental stress factors can cause dysbiosis and diarrhea in young mammals (Karasova et al., 2021). To delineate associations between gut microbes and resistance to heat stress in piglets, we employed 16S rRNA gene amplicon sequencing and included additional healthy control animals that received no treatment. The OTU rank abundance curve revealed the abundance and rarity of OTU in the fecal microbiota community of our samples regardless of treatments. All curves became gradually stable, indicating a uniform bacterial taxa distribution (Fig. 2A). The PLS-DA exhibited distinct microbiota structure separations among the healthy control, HS-RES, and HS-SUS piglets (Fig. 2B). Moreover, results of the Shannon index and inverse Simpson index consistently showed that heat stress caused a significant decrease of the intestinal microbial α-diversity in the HS-SUS piglets compared to the control and the HS-RES group (*P* < 0.05; Fig. 2C and D), while the control and the HS-RES groups were at similar levels (*P* > 0.05). Further analysis of microbiota at the phylum and family levels showed differences in bacterial community composition of the control and the HS-RES group compared to the HS-SUS group (Fig. 2E and F). In particular, at the family level, the relative abundance of Lactobacillaceae in the HS-SUS group was significantly lower than in the other groups (FDR q < 0.05). In contrast, the relative abundance of Streptococcaceae (FDR q < 0.01) and Methanobacteriaceae (FDR q < 0.05) in the HS-SUS group was significantly higher than in the other groups. In comparison, the bacterial community composition of the HS-RES animals was similar to the healthy control piglets at different taxonomic levels.

### 3.3 Identification of a *L. johnsonii* N5-associated with diarrhea resistance from heat stress-exposed piglets

**Fig. 3:**
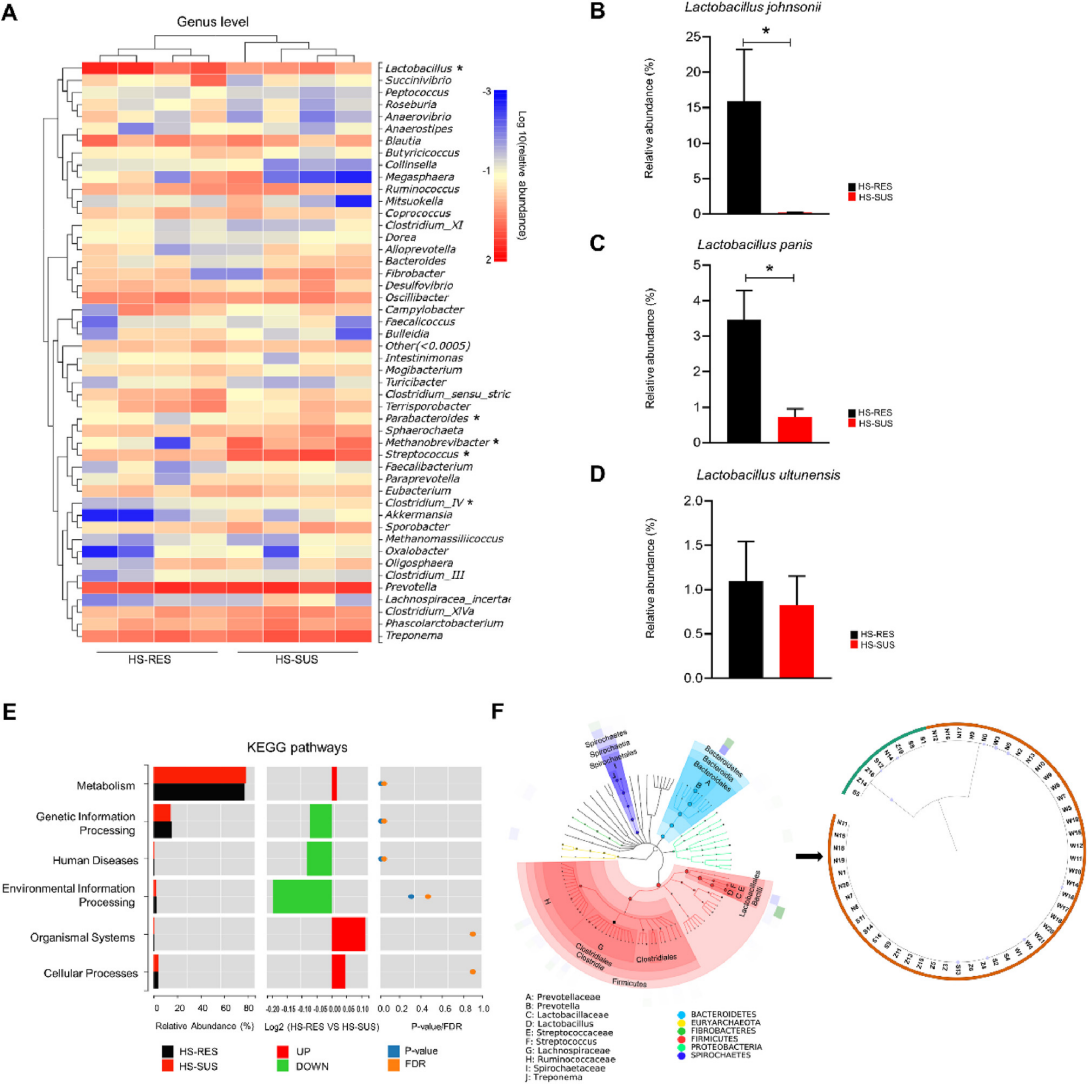
Identification of a potential probiotic *Lactobacillus johnsonii* strain associated with heat stress-resistance. (A) Heatmap of the microbiota composition in the feces from the heat stress-susceptible versus the heat stress-resistant piglets at the genus level. The top tree showed the clustering relationship of samples. In the heatmap, red color means higher relative abundance whereas blue color signifies lower relative abundance. (B to D) Relative abundance of *L. johnsonii*, *Lactobacillus panis* and *Lactobacillus ultunensis* at the species level in the HS-RES and HS-SUS group. (E) The KEGG classification of the differential microbiota functions. (F) Cladograms generated from LEfSe analysis showing the microbial clades with the greatest differences in the abundance of microbiota from the HS-RES and HS-SUS group (left panel); The distribution circles of fecal bacterium depict taxonomic assignments for the bacteria isolated from the HS-RES group and the phylogenetic tree of representative strains (right panel). HS-RES = heat stress resistant; HS-SUS = heat stress susceptible; KEGG = kyoto encyclopedia of genes and genome. ∗*P* < 0.05, using ANOVA with Tukey's post hoc test.

To identify the specific microbes that have provided resistance to heat stress-induced diarrhea and inflammation, we focused on the comparison of the microbiota between the HS-RES and HS-SUS samples. Analysis of the microbial composition at the genus level revealed an enrichment of *Lactobacillus*, and a reduction of *Parabacteroides*, *Methanobrevibacter*, *Streptococcus*, and *Clostridium_*cluster IV in the HS-RES group compared with the HS-SUS group (FDR q < 0.05; Fig. 3A). Moreover, we analyzed the 3 identified *Lactobacillus* species in the fecal microbiota. The relative abundance of *L. johnsonii* and *Lactobacillus panis* was significantly higher in the HS-RES group, while in the HS-SUS group, these bacteria were almost undetectable (FDR q < 0.05; Fig. 3B and C). However, no significant difference was observed in the relative abundance of *Lactobacillus ultunensis* between the two groups (FDR q > 0.05; Fig. 3D). In addition, the 16S rRNA data were annotated with functional pathways from the KEGG database. Compared with the HS-SUS group, the gut microbiome distinction of piglets resistant to heat stress-induced diarrhea was reflected by the upregulated metabolism and downregulated genetic information processing, human disease, and environmental information processing pathways (FDR q < 0.05; Fig. 3E). Finally, by comparing the distribution circles of the fecal bacterium at the species level (Fig. 3F, left panel), we identified *Lactobacillus* species correlated with resistance to heat stress-induced diarrhea. Fecal microbiota is a common source of probiotic isolation. We therefore performed bacterial cultivation and sequencing and obtained 57 isolates from the pooled samples (Fig. 3F, right panel). As expected, 49 out of 57 isolates belonged to the *Lactobacillus* species. It was dominated by *L. johnsonii* strains, among which a representative strain *L. johnsonii* N5 was further studied.

### 3.4 Oral administration of *L. johnsonii* N5 confers protection against DSS-induced colitis

**Fig. 4:**
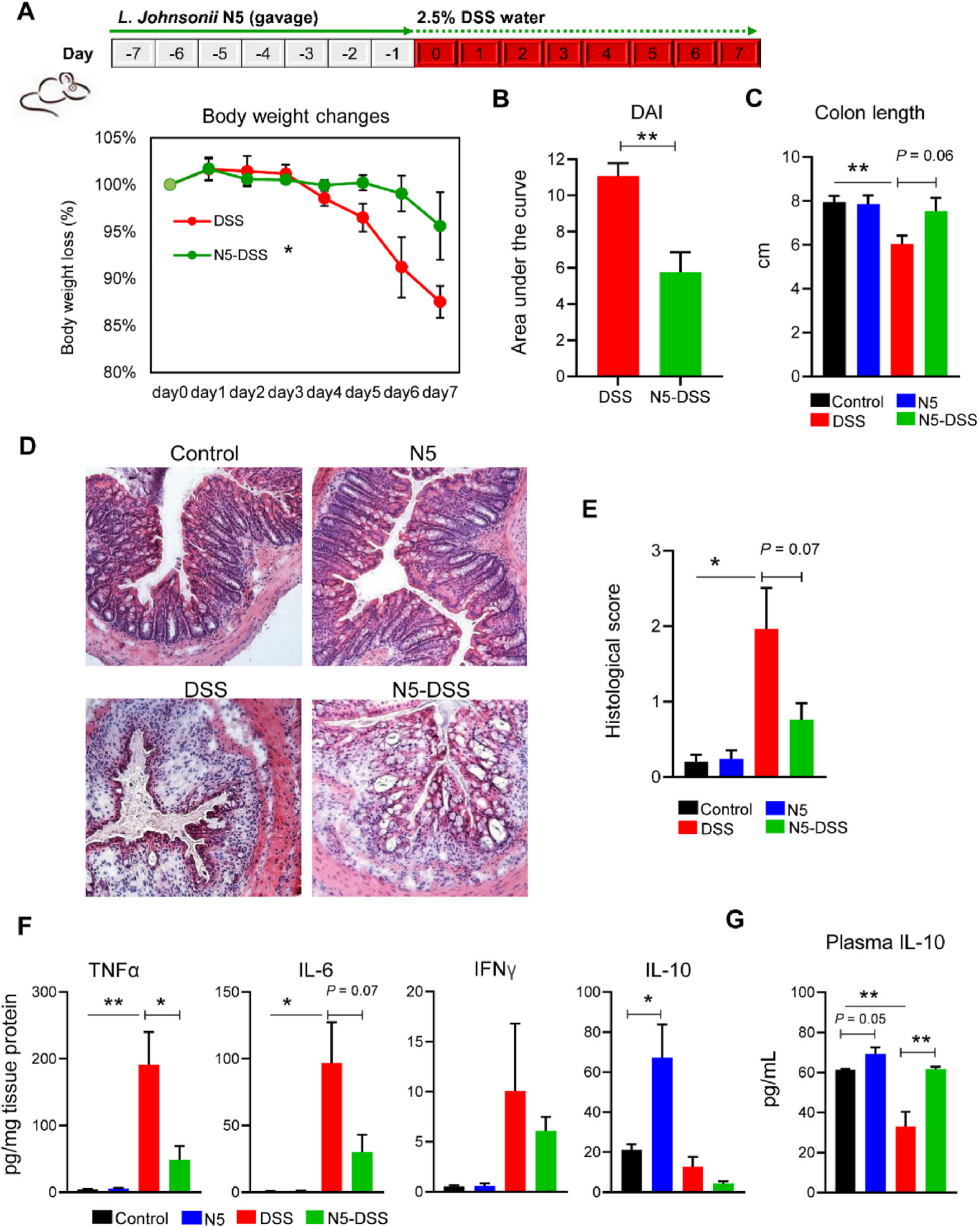
*Lactobacillus johnsonii* N5 protects against colitis in mice induced by dextran sulfate sodium. (A) Wild-type BALB/c mice were fed a commercial chow diet and received vehicle treatment (Control); or given 2.5% of dextran sulfate sodium in drinking water for 7 consecutive days (DSS); or administered *L. johnsonii* N5 daily for 7 days (N5) or 14 days starting 7 days prior to DSS-treatment (N5-DSS). Body weight changes of mice from DSS and N5-DSS group. (B) The disease activity index (DAI) was measured in mice from DSS and N5-DSS group. (C) The lengths of colon from each group. (D) Representative images of H&E-stained colonic tissue in mice from each group (10× magnification). (E) Histological scores evaluations (2 slides per mouse were analyzed). (F) Concentrations of TNF-α, IL-6, IFN-γ, and IL-10 in the colon. (G) Concentrations of IL-10 in the plasma. TNF-α = tumor necrosis factor-alpha; IL-6 = interleukin-6; IL-10 = interleukin-10; IFN-γ = interferon gamma. Data are presented as mean ± SEM, *n* = 6 mice per group. ∗*P* < 0.05, ∗∗*P* < 0.01, using ANOVA with Tukey's post hoc test.

The administration of DSS into drinking water in mice leads to diarrhea, inflammation, and intestinal tissue damage (Cooper et al., 1993; Wang et al., 2020). To study whether there were benefits to mammalian hosts following *L. johnsonii* N5 treatment, we employed the mouse model of colitis by giving a lower dose of DSS at 2.5% for 7 consecutive days (Fig. 4A, upper panel). When compared to the DSS-only group, mice that received N5 showed significantly less body weight loss (*P* < 0.05; Fig. 4A), reduced disease activities (*P* < 0.01; Fig. 4B), and a tendency to preserve the colon length (*P* = 0.06; Fig. 4C). In contrast, the N5-only treatment did not affect the colon length of mice compared to the control group (*P* > 0.05; Fig. 4C). Consistently, the histological analysis of colon tissues showed strong immune cell infiltration and mucosal damage in the DSS group, supported by an increase in histological scores in these mice compared to the control groups (*P* < 0.05; Fig. 4D and E). In contrast, pre-treatment of N5 to DSS-treated mice reduced the histological signs of inflammation and inclined to restore the histological scores (*P* = 0.07; Fig. 4D and E). No significant difference in colon histology was observed in mice not receiving DSS. In agreement with clinical and histological evaluations, the levels of TNF-α and IL-6 were significantly higher in the colonic tissue homogenates of DSS-treated mice than in the other groups (*P* < 0.05; Fig. 4F). However, no significance was detected in IFN-γ levels among treatments (*P* > 0.05). By contrast, pre-treatment of N5 was able to restore the colonic TNF-α and IL-6 levels in the DSS-treated mice. In addition, the levels of IL-10 were assessed in both colon and circulation of mice. The N5-only treatment increased its concentration compared to the healthy control mice (*P* ≤ 0.05; Fig. 4F and G), whereas the DSS treatment decreased the IL-10 levels in plasma (*P* < 0.01; Fig. 4G). By contrast, pre-treatment of N5 preserved this parameter in the DSS-treated mice (*P* < 0.01) to a similar level as in the control group.

### 3.5 *L. johnsonii* N5 improves the intestinal barrier tight junction proteins and HSP70 expressions in DSS-induced colitis

**Fig. 5:**
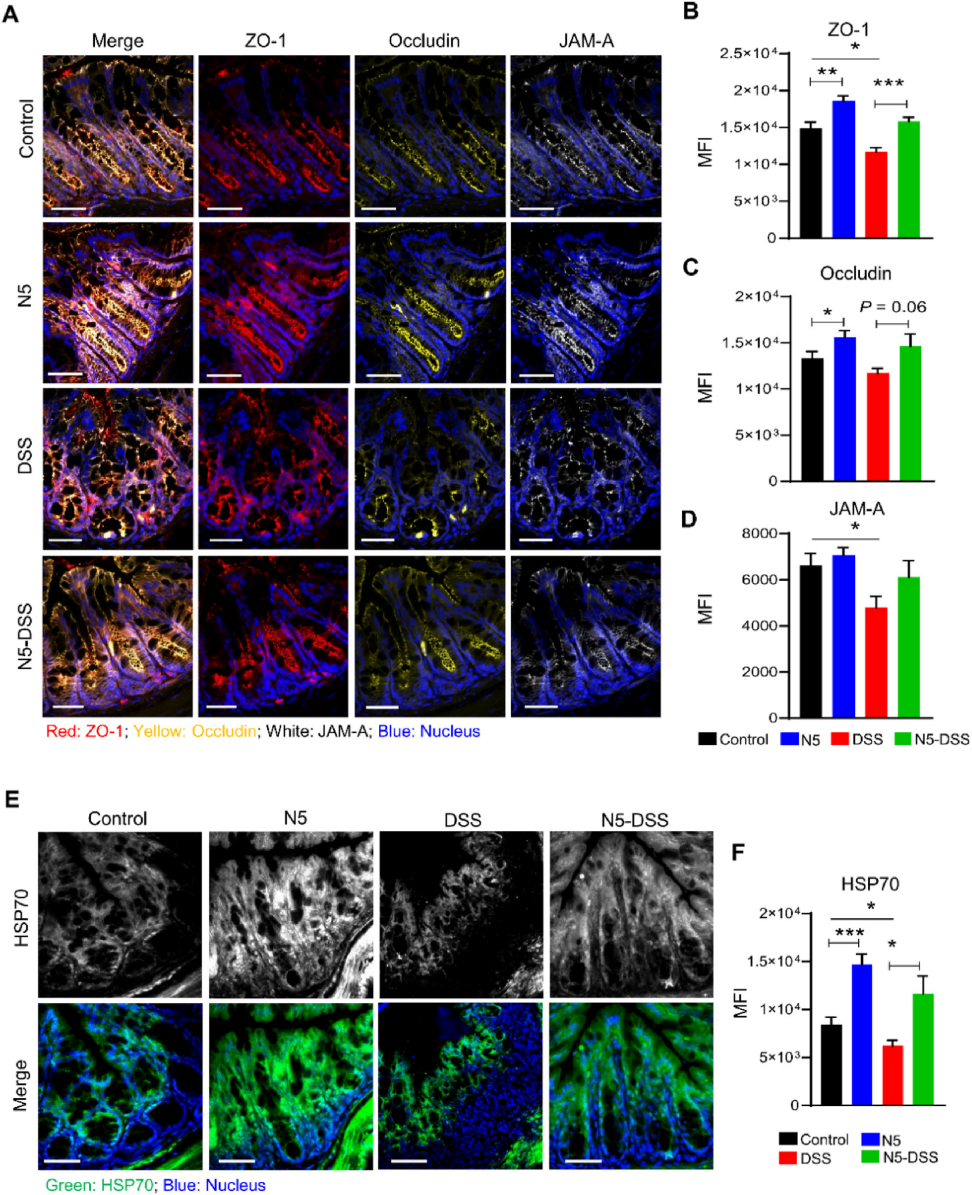
*Lactobacillus johnsonii* N5 improves the intestinal barrier tight junction protein and HSP70 expressions in dextran sulfate sodium-induced colitis. (A) Representative images of colonic tissue sections stained with antibodies specific for ZO-1 (red), occludin (yellow), and JAM-A (white). Sections were counterstained with Nucleus (blue). Scale bars = 50 μm. (B to D) The expression of ZO-1, occludin, and JAM-A was quantified as mean fluorescence intensity (MFI). (E) Representative images of colonic tissue sections stained with anti-HSP70 (green) antibody. Sections were counterstained with Nucleus (blue). Scale bars = 50 μm. (F) Analysis and quantification of HSP70 expression, *n* = 6 mice per group (duplicate slides per mouse). Control, mice were fed a commercial chow diet and received vehicle treatment; DSS, mice were given 2.5% of dextran sulfate sodium in drinking water for 7 consecutive days; N5, mice were administered *L. johnsonii* N5 daily for 7 days; N5-DSS, mice were administered *L. johnsonii* N5 daily for 14 days starting 7 days prior to DSS-treatment. ZO-1 = zonula occludens protein 1; JAM-A = junctional adhesion molecule A; MFI = mean fluorescence intensity; HSP70 = heat shock proteins 70. Data are presented as mean ± SEM, ∗*P* < 0.05, ∗∗*P* < 0.01, ∗∗∗*P* < 0.001, using ANOVA with Tukey's post hoc test.

The expression of TJ proteins ZO-1, occludin, JAM-A and cytoprotective HSP70 in the colon was studied using fluorescently labeled antibodies. The DSS-induced colitis caused significantly reduced expressions of colonic ZO-1, JAM-A, and HSP70 (*P* < 0.05; Fig. 5). However, the expression levels of these proteins were restored completely in the N5-DSS group to similar levels as those in the control group. While the expression of occludin was not affected (*P* > 0.05; Fig. 5A and C). In addition, the result showed that the N5-only treatment in healthy mice enhanced the expression of ZO-1, occludin, and HSP70 compared to the control group (*P* < 0.05; Fig. 5).

### 3.6 *L. johnsonii* N5 modulates the immune homeostasis of Peyer's patches in DSS-induced colitis

**Fig. 6:**
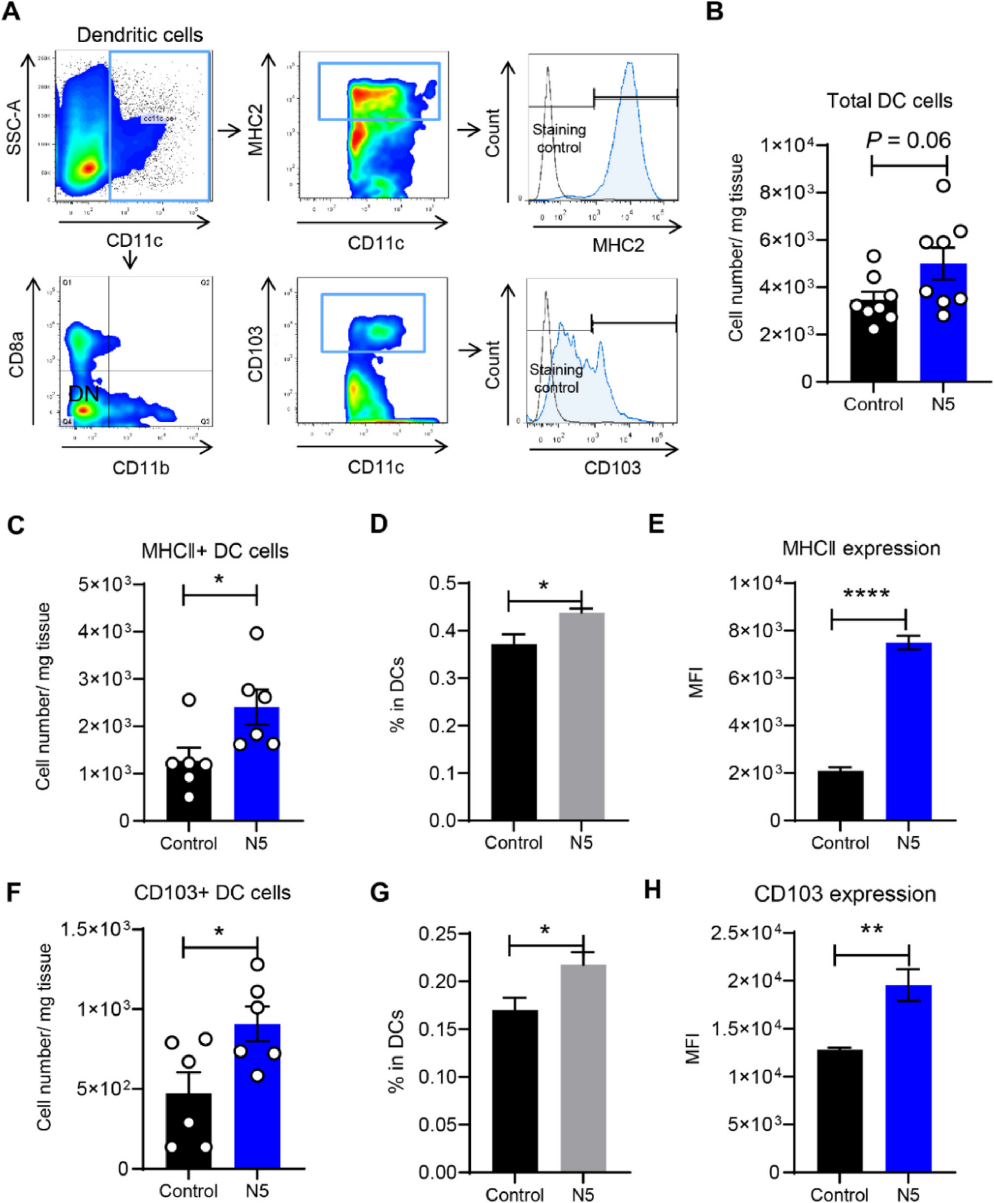
*Lactobacillus johnsonii* N5 regulates the intestinal Peyer's patches (PP) dendritic cell (DC) populations in mice. (A) The gating strategy of DC subsets isolated from PP for flow cytometry analysis. (B) The total DC cell numbers in PP. (C to E) The numbers and percentage of MHCII^+^ DC cells in PP, and the MHCII expression (MFI). (F to H) The numbers and percentage of CD103^+^ DC cells in PP, and their CD103 expression (MFI). Control, mice were fed a commercial chow diet and received vehicle treatment; N5, mice were administered *L. johnsonii* N5 daily for 7 days. MFI = mean fluorescence intensity. Data are presented as the means ± SEM, *n* = 6 to 8 mice per group. ∗*P* < 0.05, ∗∗*P* < 0.01, ∗∗∗∗*P* < 0.0001, using the two-tailed Student's *t*-test.

Intestinal PP are the major inductive sites for bacteria-immune cell reactions and are as essential as the gut epithelium for maintaining intestinal homeostasis (Liu et al., 2021; Wang et al., 2015). Based on the findings that N5 protected against DSS-induced diarrhea, inflammation, and colonic epithelium damage, we further investigated its effects on innate immunity and the balance of Treg/Th17 in adaptive immunity. Using flow cytometry, it was showed that N5 tended to increase the total DC numbers in the PP of healthy mice compared with the control group (*P* = 0.06; Fig. 6A and B). Furthermore, we observed significant increases in the MHCII^+^ DC subset, its percentage in the total DC population, and the MHCII expression levels in these cells with N5 treatment compared with the control group (*P* < 0.05; Fig. 6C to E). Meanwhile, N5 treatment also induced the expansion of the CD103^+^ DC subset, its proportion in total DC, and the CD103 expression in these cells significantly compared to the control mice (*P* < 0.05; Fig. 6F to H).

**Fig. 7:**
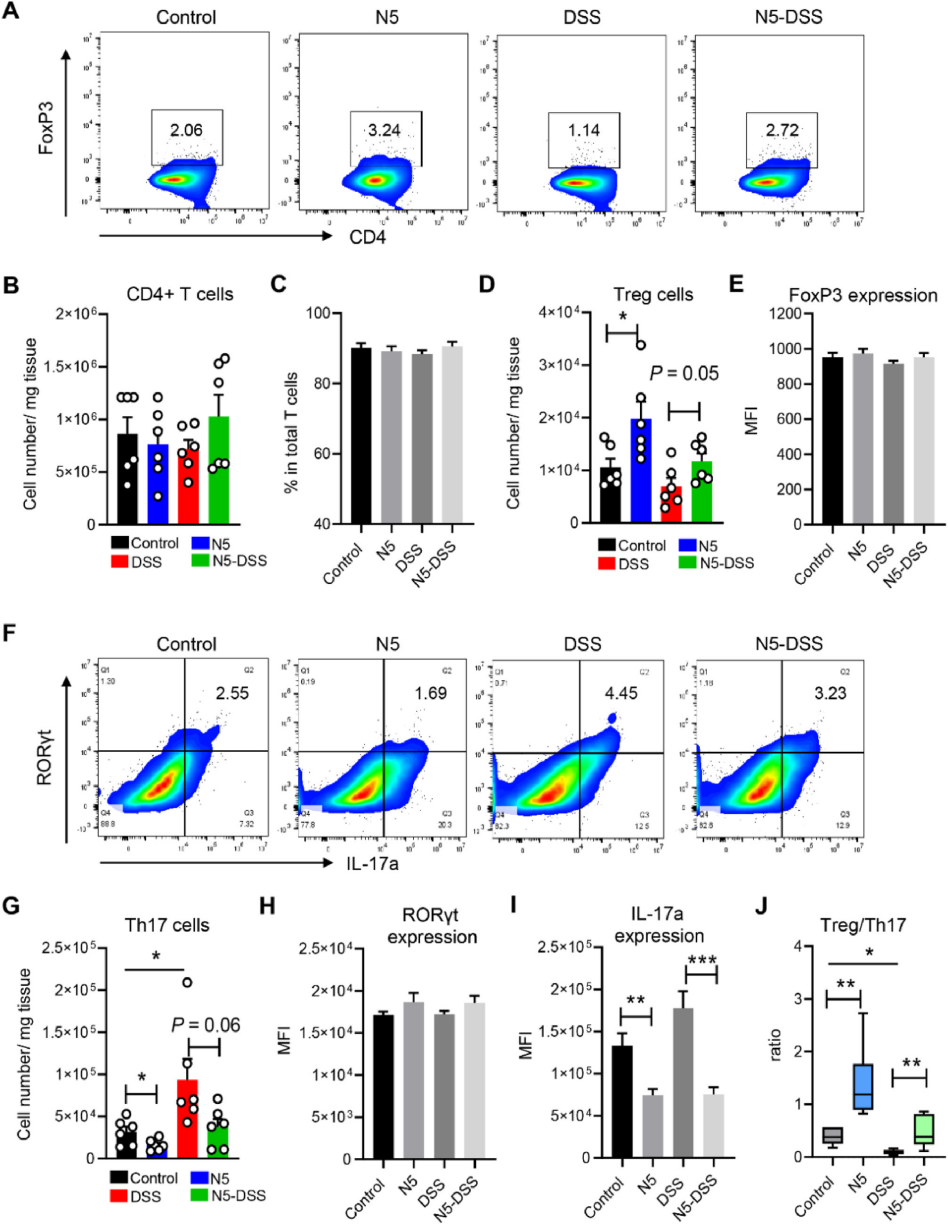
*Lactobacillus johnsonii* N5 modulates the intestinal Peyer's patches (PP) T cell responses in dextran sulfate sodium-induced colitis. (A) Representative flow cytometry profiles of FoxP3^+^CD4^+^ regulatory T cells (Treg) in PP from different treatments. (B to C) The numbers and the percentage of CD4^+^ T cells in total T cell population. (D to E) The number Treg in PP and their FoxP3 expression levels (MFI). (F) Representative flow cytometry profiles of RORγt^+^IL-17a^+^ Th17 populations in PP. (G) The number of Th17 cells in PP. (H to I) The expression levels of RORγt and IL-17a in Th17 cells in PP (MFI). (J) The ratio of Treg to Th17 cells in PP. Control, mice were fed a commercial chow diet and received vehicle treatment; DSS, mice were given 2.5% of dextran sulfate sodium in drinking water for 7 consecutive days; N5, mice were administered *L. johnsonii* N5 daily for 7 days; N5-DSS, mice were administered *L. johnsonii* N5 daily for 14 days starting 7 days prior to DSS-treatment. FoxP3 = forkhead box protein P3; MFI = mean fluorescence intensity; Treg = regulatory T cells; RORγt = retinoic acid-related orphan receptor gamma t. Data are presented as the means ± SEM, *n* = 6 mice per group. ∗*P* < 0.05, ∗∗*P* < 0.01, ∗∗∗*P* < 0.001, using ANOVA with Tukey's post hoc test.

Finally, we demonstrated that N5 treatment specifically increased the FoxP3^+^ Treg population in the PP of healthy mice and in the N5-DSS group (*P* ≤ 0.05; Fig. 7A and D; gated as shown in Fig. S1) without altering the expression of its transcription factor FoxP3. Nor did it affect the total CD4^+^ T cell numbers and their percentage (*P* > 0.05; Fig. 7B, C, and E). Contrary to the Treg responses, the DSS-only treatment increased the number of Th17 cells (RORγt^+^IL-17a^+^CD4^+^) compared to the control group (*P* < 0.05; Fig. 7F and G). While the expressions of its transcription factor RORγt or the effector molecule IL-17a were not different in the DSS-treated group from the control (*P* > 0.05; Fig. 7H and I). Interestingly, pre-treatment of only N5 decreased the number of Th17 cells and their IL-17a production in PP compared to the health control group and pre-treatment of N5 reduced these parameters in DSS-induced colitis (Fig. 7G and I). The changes of Treg and Th17 in PP resulted in a significantly higher ratio of Treg/Th17 in the N5 group than the control group and higher in the N5-DSS group compared to the DSS-only group (*P* < 0.01; Fig. 7J). In contrast, DSS treatment decreased this ratio compared to the control mice (*P* < 0.05).

## 4 Discussion

Given that the host phenotypes can be associated with intestinal microbiota, we explored the potential of *Lactobacillus* from weaned piglets resistant to diarrhea, demonstrating its protective roles in health and diseases. Here, we found significant differences between the heat stress-resistant and heat stress-susceptible individuals regarding improved growth performance, diarrhea index, and intestinal and systemic inflammatory responses. By comparing and linking the gut microbiome distinction of the two, we identified a *L. johnsonii* strain N5 and conferred its protection against DSS-induced colitis. Oral administration of N5 reduced the clinical and histological signs of colonic injury in DSS-challenged mice while preserving expressions of the intestinal TJ proteins and cytoprotective HSP70. Meanwhile, we have also demonstrated that N5 could modulate the intestinal PP DC responses and restore the Treg/Th17 immune balance. Our study has expanded the understanding of *Lactobacillus*-based probiotics in gut health management and suggests that N5 may be a promising antibiotic alternative for preventing diarrhea in animals.

Among various environmental stressors inducing diarrhea and affecting animal productivity, heat stress is one major challenge that causes growth impairment, inflammation and consequently high economic losses in pig production (Hu et al., 2022; Ringseis and Eder, 2022). These were also observed in piglets exposed to chronic heat stress during nursery in the current study. However, some individuals (i.e., HS-RES) did not show signs of diarrhea at all, in contrast to some (i.e., HS-SUS) that exhibited high diarrhea index, significant body weight loss, and increases in TNF-α, IL-6, and CXCL1 in circulation. In mammals, systemic inflammation is a hallmark of intestinal barrier disruptions in response to heat stress (Leon and Helwig, 2010; Ringseis and Eder, 2022). In the current study, we reported a marked reduction of colonic HSP70 expression at both mRNA and protein levels in the HS-SUS piglets compared to the HS-RES ones, with a down-regulation of its transcription. Heat shock proteins are intestinal gatekeepers carrying out essential cytoprotective and immunomodulatory functions against various stimuli (Liu et al., 2014a). It suggests that reducing HSP70 levels contributes to the severity of chronic IBD (Wang et al., 2018b), whereas an increase of HSP70 expression is observed in the ileum of heat stress-exposed piglets. However, in their experiment, it is acute high-temperature exposure over 3 days (Gabler et al., 2018) in contrast to the 8 weeks' chronic heating treatment in our study, which is a more realistic model of sub-tropical and temperate region summer (Ringseis and Eder, 2022). Nevertheless, the reduction of colonic HSP expression in the current study may be a result of exhaustion when the gut of HS-SUS piglets depleted the capacity to adapt to heat stimuli. In addition, gut microbiota dysbiosis or resilience also determines the intestinal barrier integrity during environmental stress (Karasova et al., 2021), as our observations in the HS-SUS versus the HS-RES piglets. Indeed, we have identified the gut microbiome distinctions: a shift of microbiota structure with a reduced community diversity; a significant loss of *Lactobacillus* genus especially the *L. johnsonii* species; and increases in abundance of *Parabacteroides*, *Clostridium* cluster IV, *Methanobrevibacter* and *Streptococcus*. The latter two are considered pro-inflammatory pathobionts in IBD (Heidarian et al., 2017; Samuel et al., 2007). Moreover, it is demonstrated that *Methanobrevibacter* (Archaea) exhibits robust cell adherent ability to the mucosa and the gut-associated lymphoid tissue (Samuel et al., 2007) and can stimulate DC to release high amounts of TNF-α and IL-1β (Bang et al., 2014). In accordance, our KEGG pathway analysis revealed metabolism alteration and upregulations of human disease and environmental information processing in the microbiota of the HS-SUS piglets, implying a disrupted intestinal microenvironment. In addition to a better understanding of the gut barrier dysfunction in the HS-SUS animals, we also demonstrated that the presence of *L. johnsonii* in the HS-RES piglets is a key component of their microbiota resistance to heat stress-induced diarrhea. This is supported by several studies showing the reduction of Lactobacillaceae and its genus *Lactobacillus* in the gut of ailing pigs in responses to environmental stress (Hu et al., 2018, 2022; Le Sciellour et al., 2019; Xia et al., 2022).

Based on the results from our study and others, we hypothesized that supplementation of *Lactobacillus* might be an effective strategy to combat diarrhea, inflammation, and intestinal barrier defects in animals. Indeed, in the current study, we demonstrated that peroral treatment of *L. johnsonii* N5 protected animals against DSS-induced colitis, including reduced signs of intestinal damage and inflammation. More importantly, evaluations of colonic barrier function showed that N5 enhanced TJ proteins and HSP70 levels in healthy mice and restored their mucosal expressions in colitis. An integral role of cytoprotective HSP70 and TJ proteins in maintaining intestinal integrity has also been shown in vitro, where inhibition of HSP concurs with TJ disruptions in Caco-2 cells in response to heat stress (Dokladny et al., 2006). In vivo, epithelial-specific expression of HSP70 rescues HSP70^−/−^ mice from DSS-induced colitis and its interaction with ZO-1 (Wang et al., 2018b). Our current findings and previous studies (Liu et al., 2014b, 2022) therefore proved that epithelial HSP70 regulation might be a shared mechanism by which probiotic *Lactobacillus* protects mammals from diarrhea and associated-gut disorders. Interestingly, although not measured directly in the current study, it is demonstrated that HSP70 also carried out immunomodulatory functions by maintaining IL-10 production (Giuliano et al., 2011; Liu et al., 2014a; Wang et al., 2018b). The production of IL-10 of DC, CD4^+^ T cells, and B cells from HSP70^−/−^ mice was diminished in association with impaired extracellular signal-regulated kinase (ERK) phosphorylation (Wang et al., 2018b).

The current study has also showed that *L. johnsonii* N5 regulates both innate and adaptive immunity of intestinal PP in addition to the HSP70-mediated probiotic effects. In particular, we found an increased population of PP DC cells in mice with *L. johnsonii* N5 pre-treatment, resulting from MHCII^+^ and CD103^+^ DC subsets expansions. Dendritic cells are widely distributed within the intestine, supporting the intestinal barrier homeostasis (Muzaki et al., 2016). Its maturation depends on the MHCII expression, and *L. johnsonii* is demonstrated to increase MHCII levels on the porcine monocyte-derived DC (Dalod et al., 2014; Zheng et al., 2021), consistent with our observations here. Furthermore, *L. johnsonii* N5 was also shown to upregulate the CD103 signaling on DC in the current study. CD103^+^ DC are crucial for propagating immune tolerance through migration to draining lymph nodes to induce gut-homing Tregs (Schnell et al., 2023). It indicates that *L. johnsonii* N5-promoted balance of DC activation, and tolerance in the gut may further shape the host T cell immunity.

In support of this, a high level of the anti-inflammatory cytokine IL-10 was observed in mice with *L. johnsonii* N5 pre-treatment at both systemic and mucosal sites. Moreover, we showed that *L. johnsonii* N5 treatment balanced the intestinal PP Treg/Th17 responses by increasing Treg cell numbers and suppressing the Th17 population and its IL-17a production under physiological condition, as well as during colitis. Tregs have been demonstrated to suppress intestinal inflammation by inhibiting IL-17 immunity in an IL-10-dependent manner (Wang et al., 2015). Similarly, He and co-workers (2019) have shown in a mouse model of autoimmune disease that *L. reuteri* treatment co-ordinately improved the gut microbiota dysbiosis and Th17 immune responses. *L. johnsonii* and its metabolites are shown to increase IL-10 secretion by DC and promote Treg cell polarization (Zheng et al., 2021). Direct inhibition of Th17 differentiation is reported in mice treated with microbiota metabolites 3-oxo-lithocholic acid (3-oxo-LCA) and iso-LCA, by binding to its master transcription factor RORγt (Hang et al., 2019). These findings underscore the potential of *Lactobacillus* in regulating Treg/Th17 balance directly or indirectly and its importance in immune tolerance generation against inflammation (Cervantes-Barragan et al., 2017; Mohamadzadeh et al., 2005; Mu et al., 2018).

Nevertheless, there are some limitations to the current study: firstly, the colonization of N5 (isolated from piglets) in a mouse model of colitis was not studied. As resident bacteria play essential roles in maintaining health, probiotic colonization in the gut has been associated with its efficacy. However, it remains controversial whether colonization is a prerequisite or if it comes with risks (Ferreiro et al., 2019); secondly, the strain specificity of N5 among bacterial taxa should be tested for general probiotic claims; finally, to confirm N5's application in livestock production, pig trials should be carried out, e.g., in preventing post-weaning diarrhea.

## 5 Conclusion

In conclusion, we have identified *L. johnsonii* N5-associated diarrhea resistance in heat stress-exposed piglets and validated its efficacy using 16S rRNA sequencing, selective culture, and supplementation experiments in an animal model of colitis. Our results demonstrated that *L. johnsonii* N5 enhances intestinal barrier function and mediates the Treg/Th17 immune balance, consequently ameliorating inflammation and preventing diarrhea. Given that antibiotics have detrimental effects on the host's health and the overall ecology, the current study provides *L. johnsonii* N5 as an alternative to manage gut disorders.

## Author contributions

**Hao-Yu Liu**, **Demin Cai:** Organized the experiment and gave some advice on experiment idea. **Long Yuan**, **Chuyang Zhu**, **Fang Gu:** Conducted the animal experiment and wrote the manuscript. **Miaonan Zhu**, **Jiacheng Yao**, **Cuipeng Zhu**, **Shicheng Li**, **Kun Wang:** Conducted the experimental analysis. **Ping Hu**, **Yunzeng Zhang**, **Hao-Yu Liu**, **Demin Cai:** Reviewed the manuscript and gave some advice on experiment idea. All authors read and approved the final manuscript.

## Declaration of competing interest

We declare that we have no financial and personal relationships with other people or organizations that can inappropriately influence our work, and there is no professional or other personal interest of any nature or kind in any product, service and/or company that could be construed as influencing the content of this paper.
