# Supplementary material for: Lactobacillus johnsonii N5 from heat stress-resistant pigs improves gut mucosal immunity and barrier in dextran sodium sulfate-induced colitis
Source: Anim Nutr. 2023 Aug 4;15:210–24. doi: 10.1016/j.aninu.2023.04.012 (PMC10685162; doi:10.1016/j.aninu.2023.04.012)
Supplement: Multimedia component 1 [file mmc1.docx]

**Table S1** Real-time PCR primer sequences.

| Name | Primer sequences (5’-3’) |
| --- | --- |
| *Hspa1a* | F: CCAATGGCATCCTGAGTGTGACAG  R: ACGAACCATCCTCTCCACCTCTTC |
| *Hspb1* | F: CGGAATTCATGGCCGAGCGCCGAGT  R: CCGCTCGAGTTACTTGTTTTCCGGCTGTTCG |
| *HSF1* | F: GCAGCAGAAAGTCGTCAAC  R: GGCGTCGTTCAGCATC |
| *GAPDH* | F: ACATCATCCCTGCTTCTACTGG R: CTCGGACGCCTGCTTCAC |

*
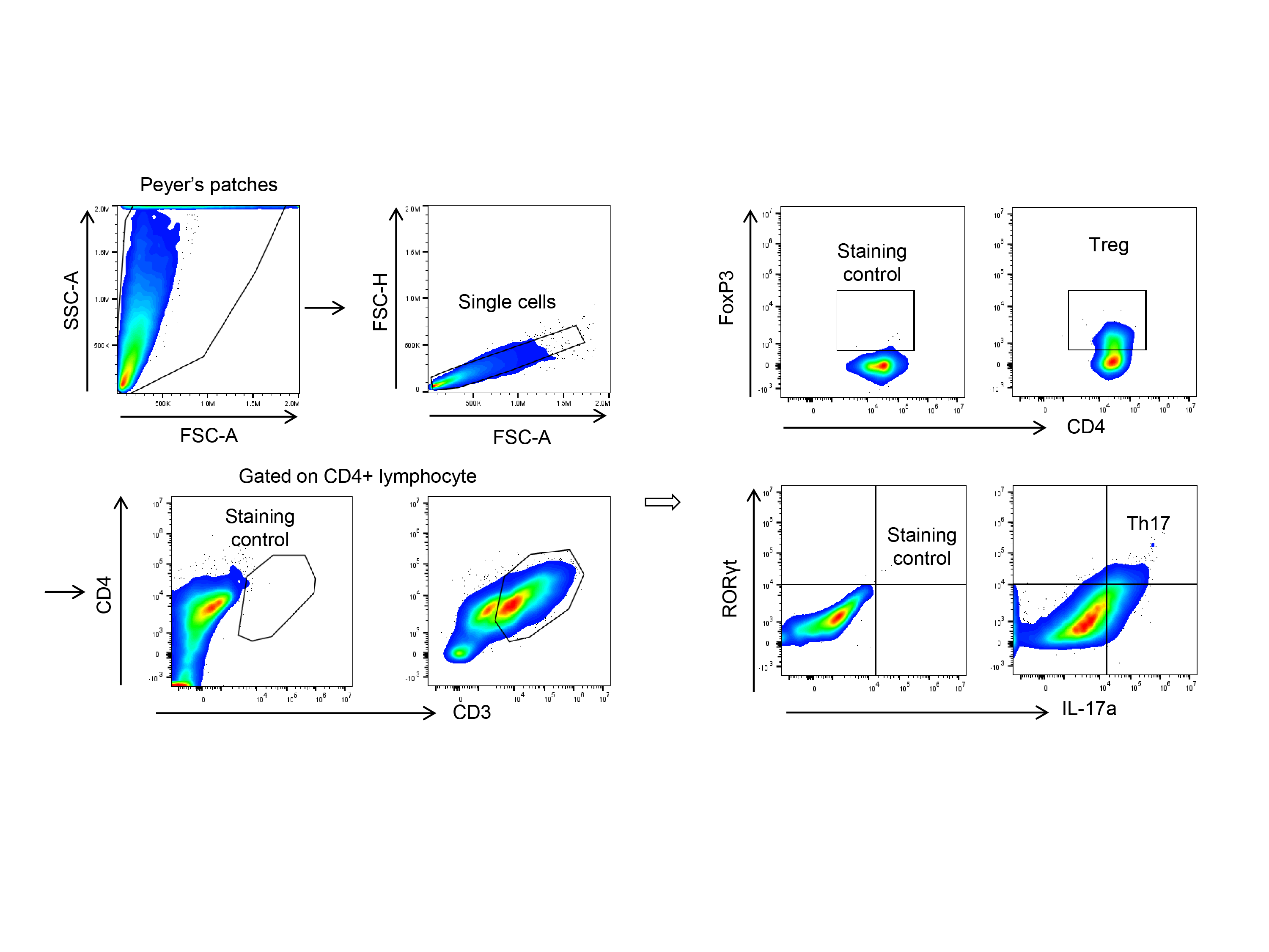
Hspa1a* = heat shock protein family A (HSP70) member 1A; *Hspb1* = heat shock protein family B (small) member 1; *HSF1* = heat shock factor 1; *GAPDH* = glyceraldehyde-3-phosphate dehydrogenase.

**Fig. S1.** The gating strategy of T cell subsets isolated from Peyer’s patches in flow cytometry analysis. SSC-A = side scatter area; FSC-A = forward scatter area; FSC-H = forward scatter height; FoxP3 = forkhead box protein P3; Treg = regulatory T cells; RORγt = retinoic acid receptor-related orphan receptor gamma t.
